# Growth and anisotropy of transport properties of *CuGaSe*_2_ single crystals

**DOI:** 10.1016/j.heliyon.2018.e00952

**Published:** 2018-11-21

**Authors:** M. Mobarak, M. Ashari, M.M. Nassary, S.G. Fatma

**Affiliations:** a Physics Department, College of Science, Jouf University, P.O. Box: 2014, Sakaka, Saudi Arabia; b Physics Department, Faculty of Science, South Valley University, Qena 83523, Egypt

**Keywords:** Condensed matter physics, Electromagnetism, Materials science

## Abstract

Single crystals of $CuGaSe_{2}$ are prepared by a technique based on the vertical Bridgman procedure. The crystal chemical and phase compositions were identified by using dispersive X-ray fluorescence spectrometry and X-ray diffraction data analysis, respectively. The Hall effect and the electrical conductivity were determined in terms of temperature, parallel and orthogonal to the layer surface, and the parameters proved to be strongly anisotropic. From carried out measurements, different parameters such like the carrier mobilities, the carrier concentration, the relaxation time, the diffusion coefficient, and the length of diffusion for both, majority carriers and minority carriers were estimated.

## 1 Introduction

The triple compound $CuGaSe_{2}$ (CGSe) belongs to the $I\text{–}III\text{–}VI_{2}$ chalcopyrite semiconductors [1]. This family of materials has got a lot of concern because they show promise for interesting practical applications [2] in photo-voltaic solar cells [3], [4], light-emitting diodes [5], and various non-linear devices [6]. Further, a considerable amount of theoretical and experimental works were done to achieve a good understanding of the optical, electronic, and electrical properties of these compounds [7], [8], [9], [10], [11], [12], [13], [14], [15], [16], [17], [18], [19]. However, the transport investigations of anisotropic properties of these compounds are scarce [20], [21]. The present report characterizes the production of $CuGaSe_{2}$ in single crystal shape by utilizing a vertical Bridgman–Stockbarger procedure. The related research works were accomplished for different materials [22], [23], [24], [25], [26], [27], [28]. Energy dispersive X-ray fluorescence spectrometry technique (EDXRF) was used to analyze the chemical composition of the $CuGaSe_{2}$ samples. The essential structural properties of $CuGaSe_{2}$ compounds were identified by X-ray diffraction analysis. However, to our knowledge, some parameters are not quite known for $CuGaSe_{2}$ crystals, such as its transport parameters and their temperature dependence. In the present study, we report the effect of the temperature on the anisotropic electrical conductivity and Hall effect for the $CuGaSe_{2}$ crystals. We also investigated the thermoelectric power measurements (TEP) of the grown $CuGaSe_{2}$ single crystals. This work is a continuation of previous works implemented for different materials [22], [29].

## 2 Experimental

**Figure 1:**
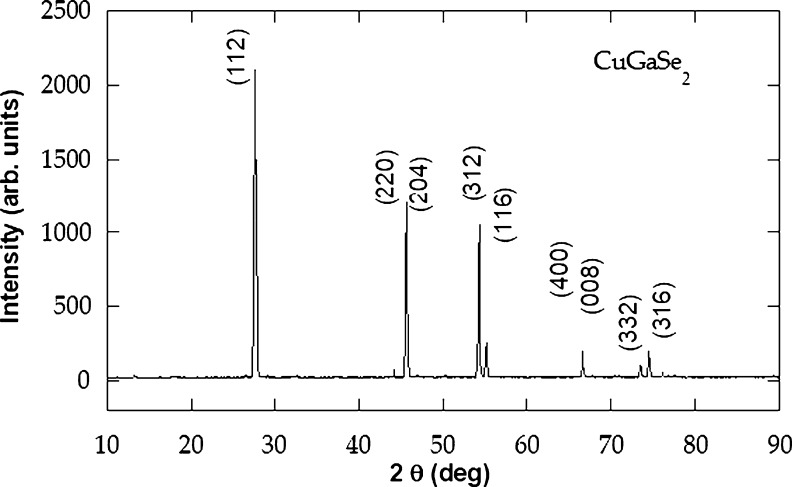
The X-ray diffraction motif recorded for *CuGaSe*_2_ powder.

The crystal growth technique used in this work was the vertical Bridgman– Stockbarger method as described in our previous papers and other works [22], [24], [29], [31], [32], [33]. Elementary copper (Cu), gallium (Ga) and selenium (Se) were used as starting materials. The needed concentrations were 24.899, 24.799 and 50.299% for Cu, Ga and Se, respectively. The mixture was kept at a melting temperature of 1030 ^∘^C for 1 day to complete the reactivity and to homogenize the melt [29]. The melting point is obtained from the known phase diagram [30], [34]. The first step after growth was to identify the crystals by using X-ray diffraction analysis and EDXRF techniques. The quantitative analysis was conducted by comparison with known standards, and the results are given in Table 1. The X-ray diffraction data assured a single-phase tetragonal (chalcopyrite structure) of $CuGaSe_{2}$ with cell parameters a=5.500±0.004 *Å* and c=11.052±0.003 *Å* with c/a=2.0095±0.0009. The energy dispersive X-ray microanalysis obviously indicated that the prepared crystals were of a stoichiometric compound corresponding to $CuGaSe_{2}$. There is a good agreement for our results with the data accessible in the literature [34], [12]. In Figure 1, the X-ray diffraction outcome for our sample is illustrated. A specimen of (9.10×2.95×2.10) $mm^{3}$ dimensions is prepared from the grown ingot using a gentle cleavage [29].

**Table 1:** Elemental analysis EDXRF data of *CuGaSe*_2_ single crystals.

Element	Weight (Wt %)	Atomic (At %)
Cu	23.12	26.38
Ga	24.78	25.77
Se	52.10	47.85
Total	100.00	100.00

The Hall effect, the electrical conductivity (*σ*) and the thermoelectric power (TEP) were also discussed in details in our previous studies [22], [24], [29].

## 3 Results and discussion

**Figure 2:**
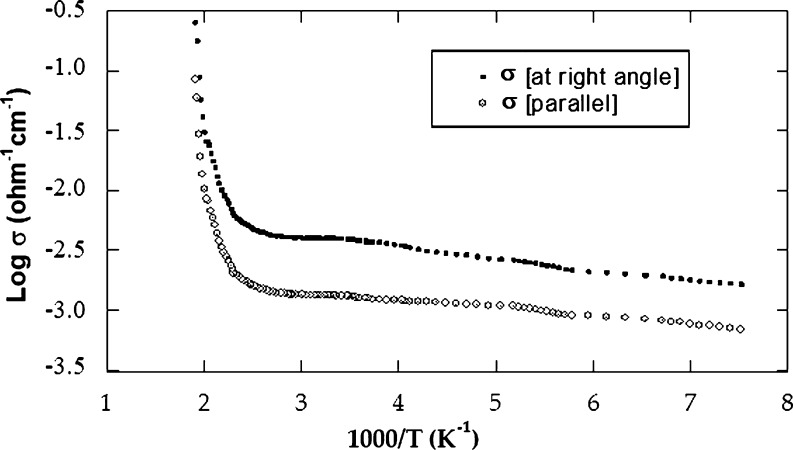
The electrical conductivity versus the temperature, as measured for *CuGaSe*_2_ crystal.

**Figure 3:**
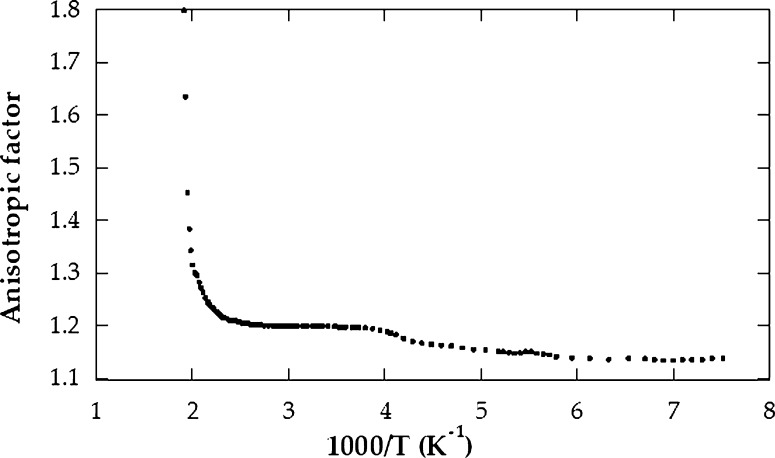
The anisotropic factor *σ*^⊥^, *σ*^//^ against temperature.

**Figure 4:**
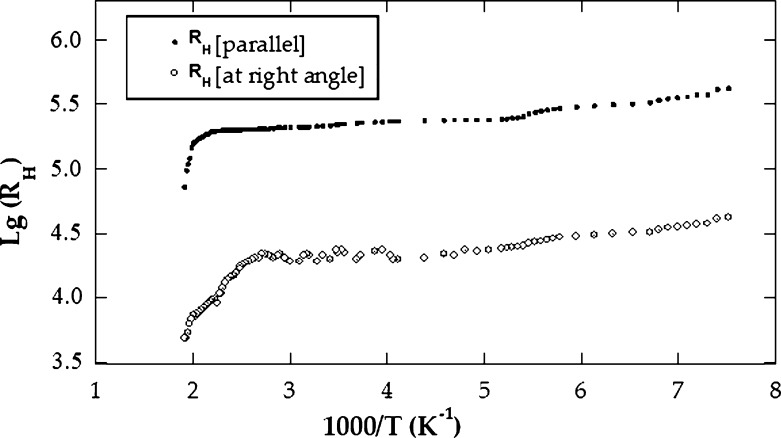
The Hall coefficient against the temperature for *CuGaSe*_2_ single crystals.

**Figure 5:**
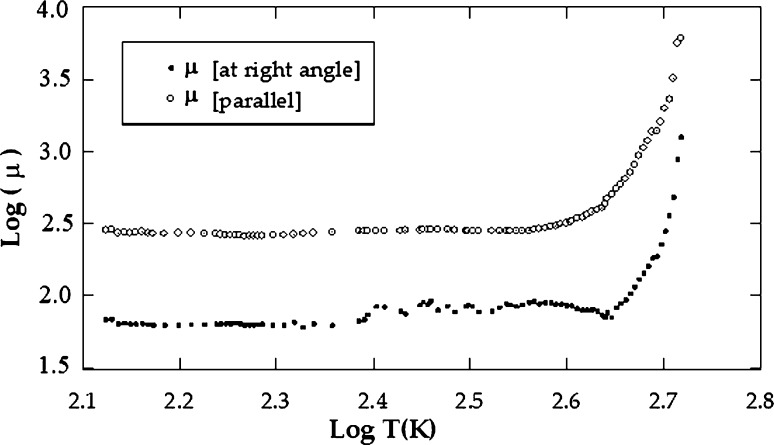
Reliance of Hall mobility on temperature for *CuGaSe*_2_.

**Figure 6:**
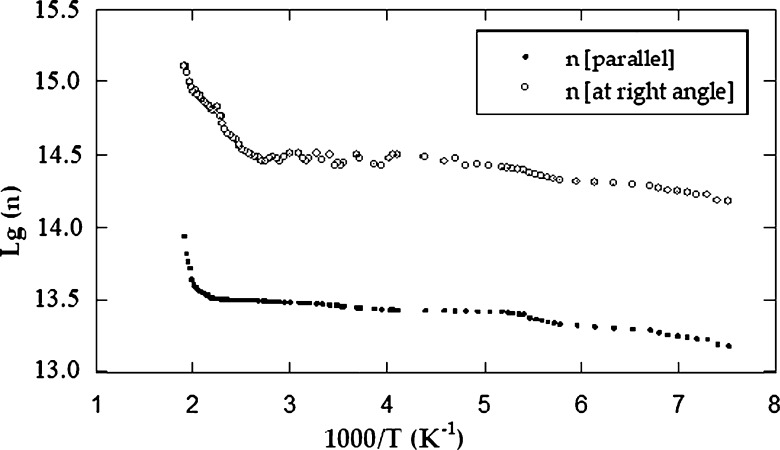
Variation of charge carriers concentration versus temperature for *CuGaSe*_2_.

In addition to direction parallel to the crystallographic c axis, the electrical conductivity *σ* is also measured in a vertical direction. Assuming that J, c and H are the current density, the crystallographic c axis and the strength of magnetic field, respectively, then the conditions of the measurements can be expressed as (J ⊥ c // H) and (J // c ⊥ H). The measurements were done over a temperature interval extending from 125 up to 530 K. Figure 2 demonstrates log *σ* Vs. 10^3^/T along and across the c axis for $CuGaSe_{2}$ single crystals. As confirmed, in the targeted temperature interval, the logarithm of *σ* demonstrated a linear reliance on temperature with two modes of conduction in addition to the transition area that revealed between them. consequently, in such semiconductor, the low-temperature side (extrinsic conduction side) appeared in the temperature interval from 125 to 260 K for $\sigma^{⊥}$ and from 125 to 290 K for $\sigma^{//}$. With regard to this extrinsic part, *σ* increased slowly with temperature. This is due to the transition of the carriers from the impurity donor band. The values of ionization energy $\Delta E_{d}$ for $CuGaSe_{2}$ were found to be 0.12 and 0.13 eV for $\sigma^{//}$, and $\sigma^{⊥}$, respectively. The transition region appeared in the interval 260 to 380 K for $\sigma^{⊥}$ and 290 to 420 K for $\sigma^{//}$. On the high-temperature side (intrinsic conduction side), it appeared in the temperature interval 380 to 530 K for $\sigma^{⊥}$ and 420 to 530 K for $\sigma^{//}$. In this area, as the temperature increases, the conductivity increases quite rapidly because of the sharp increase in the total electric current density (electrons plus holes) [22], [29]. The energy gap width $\Delta E_{g}$ for $CuGaSe_{2}$ was found to be 1.72 eV for $\sigma^{⊥}$ and 1.71 eV for $\sigma^{//}$; these outcomes are in good agreement with old data [24], [9]. For instance, the electrical conductivity at 27 ^∘^C is equal $3.30\times 10^{-3}$ (Ω${\;cm)}^{-1}$ for $\sigma^{⊥}$ and $1.33\times 10^{-3}$ (Ω${\;cm)}^{-1}$ for $\sigma^{//}$. It is obvious from the curves shown in Figure 2 that the conductivity in the direction orthogonal and parallel to c axis are strongly different, indicating a high anisotropy of $CuGaSe_{2}$ crystals. The anisotropy is characterized by the factor $N=\frac{\sigma^{⊥}}{\sigma^{//}}$ where $\sigma^{//}$ and $\sigma^{⊥}$ are the conductivities in the direction parallel to the crystallographic c axis of the crystal and orthogonal to it, respectively, and has a value 2.47 at 27 ^∘^C. The anisotropy ratio is noticed to be varied with temperature. The anisotropic factor in terms of absolute temperature is demonstrated in Figure 3. It is clear that $\sigma^{⊥}$ has a value larger than $\sigma^{//}$ in the investigated temperature interval. This denotes that *σ* is strongly anisotropic for $CuGaSe_{2}$ crystals. This can be assigned partly or totally to inter layer macroscopic disorders and or levels of precipitates. This also may be due to existing of the “two-dimensional defects” placed between layers and responsible for the carrier transition across the layers. This interpretation has been already anticipated in similar layer compounds [35]. Figure 4 shows the Hall coefficient against the temperature in the two directions of the single crystal. The evaluated shape of the figure is relatively identical to that acquired commonly in semiconductors. In contrast to our previous work [29], the conductivity during the entire temperature interval was found to be n-type for our $CuGaSe_{2}$ single crystal as concluded from the negative sign value of the Hall coefficient. The value of $R_{H}$ at 27 ^∘^C equals $2.28\times 10^{5}$ $cm^{3}/C$ at right angle to the layer planes and $2.12\times 10^{5}$ $cm^{3}/C$ parallel to the layer planes. On the low-temperature side, we deduced the energy of ionized donor atoms ($\Delta E_{d}$). The value of ($\Delta E_{d}$) for $CuGaSe_{2}$ was 0.134 ± 0.001 and 0.132 ± 0.001 eV across to the layer planes and along to the layer planes, respectively. From the high temperature side (intrinsic region), the forbidden gap width ($\Delta E_{g}$) was deduced. The value of $\Delta E_{g}$ for $CuGaSe_{2}$ was found to be 1.70 eV at the right angle to the crystallographic c axis and 1.69 eV parallel to it. Two regions of the curve can be distinguished. Figure 5 demonstrates the dependence of Hall mobility on temperature in both vertical and parallel directions to the c axis. The first part is below 380 K where it obeys the relation $\mu_{n}$ ≈ $T^{n}$ where n is equal to 1.25 for $\mu^{⊥}$ and 1.10 for $\mu^{//}$. This dependence implies that scattering mechanisms may be explained as a result of scattered impurity after its ionization in the extrinsic region [29]. At the high-temperature interval from 380 to 530 K, the mobility grows with temperature according to the low $\mu_{n}$ ≈ $T^{n}$ too. The mean value of the power n was evaluated to be 8.75 for $\mu^{⊥}$ and 8.50 for $\mu^{//}$. In this domain, we assume that the essential cause for the spreading mechanism is the spreading optical phonons. At 27 ^∘^C, the electron mobility value is 769 for $\mu^{⊥}$ and 283 $cm^{2}/Vs$ for $\mu^{//}$. The variation of charge carriers concentration versus absolute temperature is illustrated in Figure 6. It is noticed from the curve that, the carrier concentration increases as the temperature rises [29].

The donor atoms ionisation energy at the low-temperature range was found to be $\Delta E_{d}$ = 0.136 eV at right angle to the c axis and $\Delta E_{d}$ = 0.133 eV parallel to it. With regard to intrinsic behaviour, the following equation can be used [36]:

(1) $${n_{i}=(N_{c}N_{v})}^{1/2}\exp-(E_{g}/2KT)=C\exp-(E_{g}/2KT)$$

From equation (1), $\Delta E_{g}$ can be estimated.

**Figure 7:**
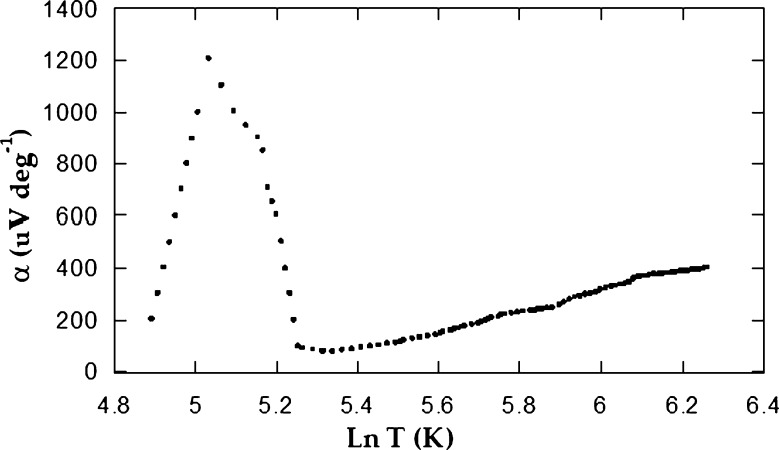
The relation between TEP *α* and natural logarithm of absolute temperature for *CuGaSe*_2_.

The energy gap width $\Delta E_{g}$ for $CuGaSe_{2}$ was found to be 1.71 eV at the right angle to the c axis and 1.70 eV parallel to it. The values are in a suitable agreement with those acquired from the conductivity and the Hall effect data. For instance, the electron concentration for $CuGaSe_{2}$ at room temperature was found to be $2.74\times 10^{14}$ $cm^{-3}$ at right angle to the cleavage planes and $2.9\times 10^{13}$ $cm^{-3}$ parallel to it. As a complementary aspect to the Hall effect and electrical conductivity, the TEP assessments were performed. The temperature tendency direction was orthogonal to the crystallographic c axis and in the temperature interval between 125 and 530 K. The outcomes demonstrate that the conductivity might be highly considered as n-type accompanied by no polarity change over the whole temperature interval, which has good agreement with Hall coefficient measurements. Figure 7 demonstrates clearly the TEP in terms of absolute temperature. From this relation, we noticed that *α* grows monotonically with temperature going through a sharp maximum value of 1208 $\mu VK^{-1}$ corresponding to 153 K. This is because of the thermal excitement of the impurity ionization in this extrinsic area. With further temperature increase, TEP falls rapidly to a minimum value 80 $\mu VK^{-1}$ at 308 K. This leads to the postulate about the attendance of trapping centres or several crystal defects in the carrier move direction. Above 308 K, *α* increases again with the temperature rise.

During high-temperature interval, *α* increases dilatory with temperature; that was interpreted before to be caused by the thermal generation of the charge carriers with rising temperature which appears in the conduction band [29]. The dependence of *α* variation on temperature in the intrinsic region is presented in equation (2).

(2) $$\alpha =\frac{K}{e}[\frac{\mu_{n}-\mu_{p}}{\mu_{n}+\mu_{p}}(\frac{\delta E_{g}}{2KT}+2)+\frac{3}{4}\ln\frac{{m^{⁎}}_{n}}{m_{p}^{⁎}}]$$

Here $\mu_{p}$, $m_{p}^{⁎}$, $\mu_{n}$ and $m_{n}^{⁎}$ are hole mobility, hole effective mass, electron mobility and electron effective mass, respectively [37], [29].

When we plot equation (2), we compute the fractions $\mu_{n}$ / $\mu_{p}$ and $m_{n}^{⁎}$ / $m_{p}^{⁎}$ from the slope-intercept form in the intrinsic region. They are 1.49 and $1.20\times 10^{-3}$, respectively. Since the quantity $\mu_{n}$ is found at room temperature as 759 $cm^{2}/Vs$, $\mu_{p}$ comes to be 509 $cm^{2}/Vs$. Another important relationship is that of Wilson [38] which is applied in the extrinsic part:

(3) $$\alpha =\frac{K}{e}[2-\ln\frac{ph^{3}}{2{\left(2\pi m_{n}^{⁎}KT\right)}^{3/2}}]$$

**Figure 8:**
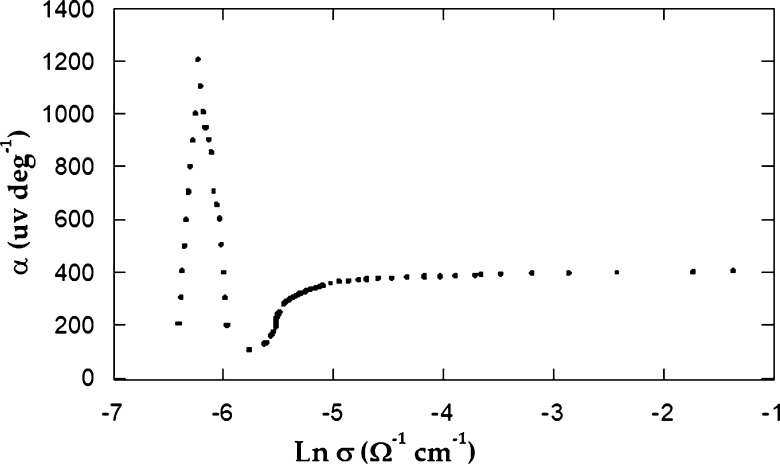
The TEP versus ln *σ* for *CuGaSe*_2_ single crystals.

When we plot equation (3), we should obtain a linear relation in the extrinsic part. From the graph, we can calculate $m_{n}^{⁎}=8.09\times 10^{-34}Kg$; hence, $m_{p}^{⁎}$ is $6.74\times 10^{-31}Kg$. Figure 8 demonstrates the reliance of TEP on the natural logarithm of *σ*, according to equation (4) [39].

(4) $$\alpha =-\frac{K}{e}[A-\ln\frac{2{\left(2\pi m_{n}^{⁎}KT\right)}^{3/2}}{{\left(2\pi h\right)}^{3}}]-\frac{K}{e}\ln\sigma$$

The relationship between *σ* and *α* is identical to the general behaviour of *α* in terms of temperature (Figure 7). The obtained effective mass values for both minority and majority charge carriers are used for the calculation of the carriers relaxation time. They are $\tau_{p}=2.10\times 10^{-16}$ and $\tau_{n}=3.80\times 10^{-19}$ s for holes and electrons, respectively. The diffusion coefficients for holes and electrons were also calculated. They were $D_{p}=12.70$ and $D_{n}=18.98$ $cm^{2}\;s^{-1}$, respectively. A further significant parameter which was evaluated from both, the relaxation time and the diffusion coefficient, was the diffusion length, because L = $\sqrt{D\tau }$. The diffusion length for electrons is $L_{n}=2.70\times 10^{-9}$ cm, while for holes, it is $L_{p}=5.20\times 10^{-8}$ cm.

The above outcomes are supposed to indicate the strong anisotropy of $CuGaSe_{2}$ crystals due to the durable anisotropy of the carrier move across and parallel to the crystal plane.

## 4 Conclusions

Single crystals of $CuGaSe_{2}$ are prepared by the modified vertical Bridgman method. The prepared crystals were identified by X-ray diffraction and energy dispersive X-ray fluorescence spectroscopy technique. The thermoelectric power, electrical conductivity and Hall coefficient were presented in terms of temperature. The Hall effect and electrical conductivity were obtained orthogonal and parallel to the layer planes for $CuGaSe_{2}$ crystals, and it proved to be highly anisotropic. From these measurements, different physical parameters were estimated in two-directional crystals. The Hall coefficient value is negative over the exact temperature interval. This indicates that the main carriers are electrons, and therefore, $CuGaSe_{2}$ crystal is n semiconductor type.

## Declarations

### Author contribution statement

M. Mobarak, M. M. Nassary, S. G. Fatma: Conceived and designed the experiments; Performed the experiments; Analyzed and interpreted the data; Contributed reagents, materials, analysis tools or data.

M. Ashari: Analyzed and interpreted the data; Contributed reagents, materials, analysis tools or data; Wrote the paper.

### Funding statement

This research did not receive any specific grant from funding agencies in the public, commercial, or not-for-profit sectors.

### Competing interest statement

The authors declare no conflict of interest.

### Additional information

No additional information is available for this paper.
